# *In-silico *prediction of blood-secretory human proteins using a ranking algorithm

**DOI:** 10.1186/1471-2105-11-250

**Published:** 2010-05-14

**Authors:** Qi Liu, Juan Cui, Qiang Yang, Ying Xu

**Affiliations:** 1 College of Life Science and Biotechnology, Tongji University, China; 2 Computational Systems Biology Laboratory, Department of Biochemistry and Molecular Biology, and Institute of Bioinformatics, University of Georgia, Athens, GA 30602, USA; 3 Department of Computer Science and Engineering, Hong Kong University of Science and Technology, Hong Kong, China; 4 College of Computer Science and Technology, Jilin University, Changchun, Jilin, China

## Abstract

**Background:**

Computational identification of blood-secretory proteins, especially proteins with differentially expressed genes in diseased tissues, can provide highly useful information in linking transcriptomic data to proteomic studies for targeted disease biomarker discovery in serum.

**Results:**

A new algorithm for prediction of blood-secretory proteins is presented using an information-retrieval technique, called *manifold ranking*. On a dataset containing 305 known blood-secretory human proteins and a large number of other proteins that are either not blood-secretory or unknown, the new method performs better than the previous published method, measured in terms of the area under the recall-precision curve (AUC). A key advantage of the presented method is that it does not explicitly require a negative training set, which could often be noisy or difficult to derive for most biological problems, hence making our method more applicable than classification-based data mining methods in general biological studies.

**Conclusion:**

We believe that our program will prove to be very useful to biomedical researchers who are interested in finding serum markers, especially when they have candidate proteins derived through transcriptomic or proteomic analyses of diseased tissues. A computer program is developed for prediction of blood-secretory proteins based on manifold ranking, which is accessible at our website http://csbl.bmb.uga.edu/publications/materials/qiliu/blood_secretory_protein.html.

## Background

Identification of disease markers in serum represents a very important problem, but it is rather challenging due to the composition complexity and the large dynamic range of proteins in human sera, which makes direct comparative analyses of serum proteomic data between diseased and control samples exceedingly difficult [1,2]. What can possibly alleviate the problem is to carry out such comparative analyses among a group of candidate protein markers rather than searching through the whole serum proteome in blind. The candidate markers could be suggested by differential analyses based on microarray gene expression or proteomic data of diseased *versus *control tissues [3]. The basic idea is to first identify genes or proteins with abnormal expression patterns in diseased *versus *control tissues, which represents a substantially simpler problem other than direct comparative proteomic analyses of serum marker identification, and then determine if the abnormally expressed proteins may possibly get secreted into blood [3-5]. The challenge in addressing the second part of the problem lies in the reality that our current understanding is rather limited about which of the expressed proteins in tissues may get secreted into blood circulation. To address this problem, we have developed the first computational method for prediction of blood-secretory proteins, using a data mining approach [6]. Specifically, a number of sequence, physicochemical and structural features of proteins, such as signal peptides, transmembrane domains, glycosylation sites, disordered regions, secondary structural content and hydrophobicity were identified, which can potentially distinguish blood-secretory from non-blood-secretory proteins. Using these features, a classifier based on support vector machine (SVM) was trained to distinguish the blood-secretory proteins from non-blood-secretory proteins.

One challenging problem in solving the so-defined blood-secretory prediction is that we did not have a clean dataset of non-blood-secretory proteins as the negative training data, a common issue encountered across many biological problems. In our previous work [6], we have taken a rather conservative approach in selecting the negative dataset by leaving out a significant fraction of proteins which could potentially be non-blood secretory proteins; hence the data may not adequately represent the whole space of the non-blood-secretory proteins.

In this study, we tackle this problem from a different perspective. We intend to *rank *the positive data out of the background data instead of to *classify *them from the rest of the background. An information retrieval technique, so called *manifold ranking *[7], was employed to rank all the candidate proteins according to the possibility of being blood-secreted, which is a semi-supervised prediction model. Its main difference from our previous classification-based approach is that it requires no negative set but only a positive set and a background set.

Ranking techniques have been successfully applied to solve various biological probelms. For example, *RANKProp *used the ranking technique for remote homology detection [7-9]. Owen et al. developed a ranking technique-based algorithm for identification of co-expressed genes [10]. *GeneRank *ranks genes in terms of their relevance to a particular stimulus based on changes in their expression levels, using a very similar idea to that of the Google *PageRank *algorithm [11,12]. *miRank *is a recent algorithm for predicting microRNAs [13], also employing a ranking technique.

It should be noted that classification techniques like SVM can also be used to rank data samples, according to their distances to the separating hyperplane in the feature space [14,15]. Nevertheless, manifold ranking has proved to be superior in this regard [7], due to its well-developed transductive ability to fully utilize the mutual relationships among the provided data. One potential drawback with the manifold ranking method, compared to an SVM, is that it is computationally more expensive, given that its computational complexity is O(*n*^3^) for a dataset of *n *samples [7,9]. In our case, *n*, the number of inclusive proteins, could be 20-30 K or larger. To deal with this computational issue, we have employed an efficient filtering procedure to reduce the initial set of candidate proteins to ensure that our predictor runs efficiently without substantially lowering the prediction accuracy.

## Methods

### A. Datasets

Two datasets were used in this study. One is consisting of 305 experimentally validated human blood-secretory proteins and 14,770 non-blood-secretory proteins [6], which were divided into a training set of 253 positive samples and 11,141 negative samples and a test set with the remaining 52 positive samples and 3,629 negative samples. Another dataset is the well-curated human proteome from *Swissprot *[16], containing 20,309 human proteins.

We continue to use the same set of features identified in our previous work [6], such as signal peptides, transmembrane domains, glycosylation sites, disordered regions, secondary structural content and hydrophobicity, by which each protein is represented as a 85-dimensional feature vector (see additional file 1 for details).

### B. A computational framework for ranking blood-secretory proteins

**Figure 1:**
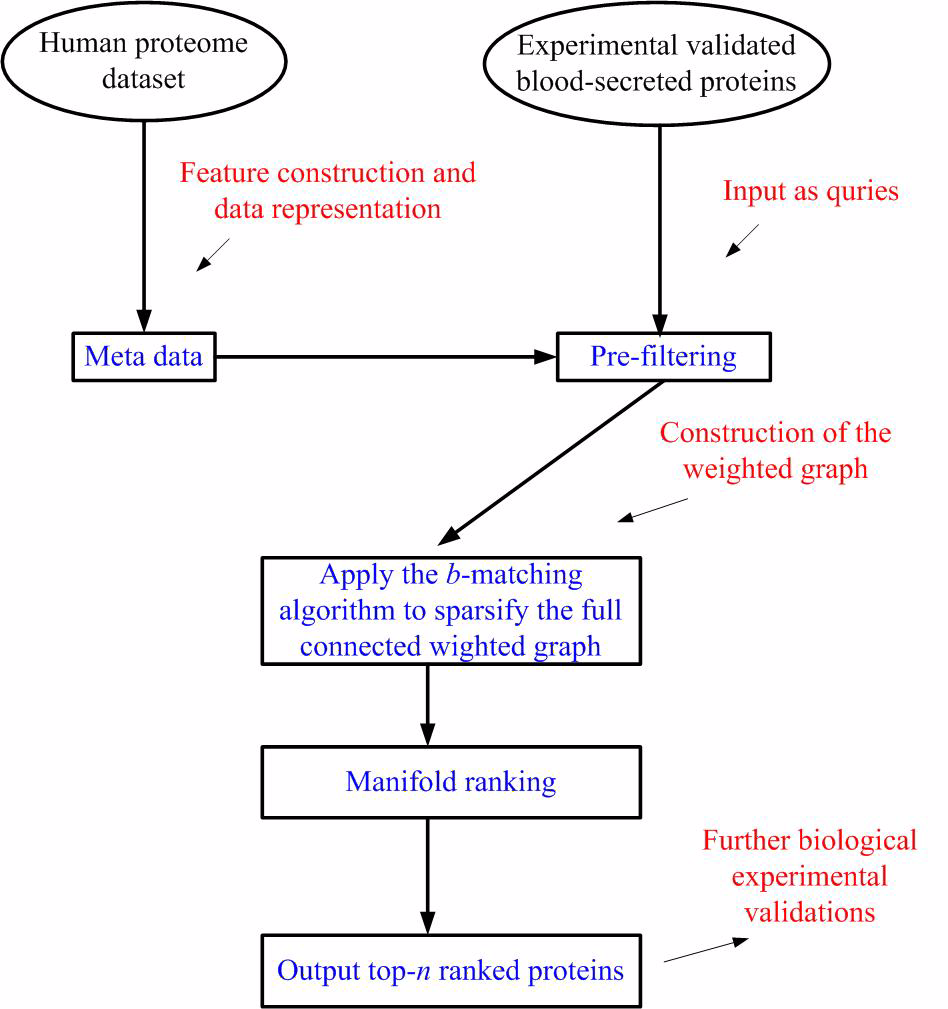
**General computational framework in our study**.

We now present a computational framework for blood-secretory protein prediction, consisting of the following steps as shown in Figure 1: (a) a pre-processing step is employed to filter out the most irrelevant proteins to the positive samples, based on the criteria described in subsection F; (b) a weighted graph is constructed as the main data structure for solving our ranking problem, based on the remaining proteins from (a). (c) This graph is sparsified with an efficient algorithm for further manifold ranking, which will be elaborated in subsection D; (d) a semi-supervised ranking algorithm is applied on the constructed graph to rank the proteins; and (d) output the *N *highest ranked proteins, where *N *is a user-specified parameter. Note that proteins with higher ranks are intended to have higher probabilities for being blood-secretory.

### C. A ranking algorithm

The essence of a manifold ranking algorithm [7,9,17] can be intuitively explained: the problem is defined on two datasets, a true sample set and an unknown sample set (background); and the goal is to rank the individual members of the unknown sample set according to their *relevance *to the true samples. A weighted graph is used to represent the whole sample set, with each sample being represented as a node of the graph and each pair of nodes being represented as an edge with a weight defined as the similarity between the two nodes in the feature space. Then an *evidence *propagation process starts, in which each true sample propagates its *presence *(as an evidence) to its neighbouring nodes to increase their relevance to the true sample set, where the increased relevance is valued proportionally to the corresponding edge weight in the graph. An overall relevance score of each node is summed over all the scores propagated to itself from all the related true samples, by which the unknown samples can be ranked eventually.

Formally, given is a set of points *X *= (*x*_1_... *x*_*q*_, *x*_*q *+ 1_... *x*_*n*_) in *R*^*m *^(*m *is the dimension of the feature space), with the first *q *points being the known blood-secretory proteins and the remaining (*n-q*) being the unknown proteins. Let *f *: *X *→ *R *be a ranking function that assigns each point *x*_*i *_a ranking score *f*(*x*_*i*_). Define *y *= [*y*_1_... *y*_*n*_]^*T *^with *y*_*i *_= 1 if *x*_*i *_is a true sample and *y*_*i *_= 0 otherwise. The aim of the ranking algorithm is to obtain a final ranking score f* for each protein through a procedure given as follows:

***Input**: A set of points X *= (*x*_1_... *x*_*q*_, *x*_*q *+ 1_... *x*_*n*_) *in R*^*m *^*with the first q points being true samples, and the initial score y*. *Define f^0 ^= y; and σ, α are two parameters of the program*.

***Output**: A ranked list of X, with higher ranked proteins being more likely to be blood-secretory proteins*.

*1. Define the edge-weight matrix (graph) W*_*ij *_= *exp*(-||*x*_*i *_- *x*_*j*_||^*2*^/*2σ*^*2*^) *and W*_*ii *_= 0. *Some of the edges can be removed using a graph sparsification procedure described in the next subsection;*

*2. Compute L = D*^-1/2^*WD*^-1/2 ^*with D being a diagonal matrix defined as ;*

*3. Set iteratively f*^*t*+1 ^= *αLf*^*t *^+ (1-*α*)*y until f converges, where α is a parameter in [0, 1);*

4. Let f* be the converged function f^t^; and rank all the points {x_i_} in the decreasing order of their f* values.

It has been shown [7] that f* converges to the following(1)

A detailed description of equation (1) is given in [9]. The two free parameters *σ *and *α *are defined as follows: *σ *defines the local affinities among all the data points while *α *controls the level of effect of the positive-sample propagation. Note that the effect of *σ *on the affinity matrix *W *will be counteracted by normalization *L *= *D*^-1/2^*WD*^-1/2 ^and will not contribute to the final ranking. In our study we have empirically set , where  is the average distance between all pair samples in a given dataset [18].

We set *α *= 0.5 based on our 10-fold cross-validation on the test dataset. To tune this parameter, we searched the optimal value of *α *from 0 to 1 with step 0.1. For each value, the positive samples in the test dataset were divided into 10 folds and the ranking is performed based on one subset as queries, and validated based on the combination of other nine subsets. This procedure was performed 10 times and the validation results are averaged over the rounds. Finally we selected the optimal value of *α *from (0,1] according to the corresponding validated ranking performance.

It should be noted that this ranking algorithm is different from sequence similarity-based algorithms such as BLAST [19], as we measure the relevance between the blood-secreted and non-blood-secreted proteins in terms of a set of biologically meaningful features.

### D. Edge removal from our graph

Note that some of the edges in the graph defined in Section C do not really contribute to the correct ranking; so we consider having such edges removed to ensure that the ranking algorithm remains efficient and robust in the presence of noise. Here, we applied a graph-sparsification method to construct a sparse graph from a fully connected weighted graph obtained from the previous steps.

Formally, for the matrix *W *∈ *R*^*n*×*n *^calculated in Section C, we first find a binary matrix *P *that maximizes the following objective function under the specified constraints:(2)

This optimization problem can be solved using a recently published algorithm, called a *loopy belief propagation *method [20]. We keep an edge in the graph defined in Section C only if its corresponding value in P is 1, and then recalculated the weights of the re-defined graph as follows:(3)

which gives a highly sparse graph without loss of the essential information for the ranking purpose [18,20]. Recent reports indicate that this sparsification procedure can be implemented efficiently using a belief propagation algorithm that ensure to obtain the global solution in cubic time of the number of nodes in the graph [18,20], which we have done.

### E. Performance evaluation

We have used the following parameter to evaluate the prediction performance of blood-secretory proteins. The *area under curve *(*AUC*) of the recall-precision curve [22] was calculated as a prediction accuracy evaluation:(4)

where TP, FP and FN are numbers of true positive predictions, false positive predictions and false negative predictions, respectively.

### F. Filtering

The computation complexity of the ranking algorithm is *O*(*n*^*3*^), where *n *is the number of samples [7], possibly causing problems when applied to large sample sizes. To overcome this issue, we have filtered out the majority of the most irrelevant unknown samples by ranking them according to their nearest distance to the known blood-secreted protein samples. We filter out *k *unknown samples from the bottom of the ranking according to such a distance, with *k *being a user specified parameter. Note that we have to calculate the pairwise distances among samples to define the affinity matrix *W *in the first step, thus additional computational time has to be introduced by this filtering step but without increasing the asymptotic computational complexity of the whole algorithm.

### G. Universal manifold ranking with both positive and negative samples

When both positive and negative training data are available, we can extend the above manifold ranking algorithm to take advantage of the availability of negative training data, giving rise to the so-called *universal manifold ranking*, an idea initially employed in [9], and we refer the above original manifold ranking algorithm based on positive training data as the *positive-samples-only-based manifold ranking *algorithm.

A high-level idea of the universal manifold ranking algorithm can be outlined as follows. When both positive and negative samples are used for training, each of the two datasets affects the overall ranking differently. In principle, positive examples should make more contributions to the final ranking than negative examples since for an unlabeled data, the farther it lies from positive examples in the feature space, the less possible it is a positive one, which in general does not have to be case for negative examples [9].

Specifically, we have two initial score vectors *y*^+ ^and *y*^-^. An element of the former is set to 1 if the corresponding sample is blood-secretory, and the element of the latter is set to -1 if the corresponding sample is not blood-secretory. All the other elements of the two vectors are set to 0. According to equation (1), we denote *A *= (1 - *a*)(*I *- *aL*)^-1 ^and define two matrices *A*^+ ^and *A*^-^, which are used to propagate the ranking scores of both the positive and negative examples, i.e., *f*^+^* = *A*^+^*y*^+^, *f*^-^* = *A*^-^*y*^-^, where *f*^+^* and *f*^-^* are the ranking scores obtained from positive and negative samples, respectively. The final ranking score can be written as:(6)

In our current implementation, we have *A*^+ ^= *A *and *A*^- ^= *γA*^+^, with *γ *∈ (0, 1] being a parameter that controls the contribution of the negative samples to the final ranking by f*.

## Results and Discussion

In this study, we continue to use the same human protein dataset from our previous work [6]. We have carried out a detailed analysis of our prediction results as follows.

### Performance on independent test data

305 experimentally validated blood-secretory proteins and 14,770 non-blood-secretory proteins have been collected. Each of these proteins is represented using an 85-dimensional feature vector. In our previous study [6], this dataset was divided into a training set with 253 positive samples and 11,141 negative samples, and a test set with 52 positive samples and 3,629 negatives. Here, we used the same test set through the following evaluation procedure to assess the comparison performance:

(1) We randomly selected 10, 20 and 30 blood-secretory proteins from the test dataset as the queries and rank all the 3,681 proteins in the test set using the *positive-samples-only-based manifold ranking *and SVM-based algorithm. We repeat this procedure five times and the average performance results were used for performance assessment. The reason for us to use three different sets of queries is obvious: we want to prove that the manifold ranking is universally superior to SVM-based ranking methods with different numbers of known positive samples. It should be noted that it is difficult to directly compare manifold ranking with SVM-based classification results, since the latter involves class labels while the former does not. To overcome the issue, we adapted two comparison strategies: (1). we followed a strategy presented by Xue et al. [21], and trained three SVM-based models with the selected 10, 20 and 30 queries as the positive training samples, respectively; and we included *N *times negative samples of the positive ones in the training set, where *N *is determined through trial-an-error to achieve the best classification results for the whole test dataset. Then the whole test proteins were ranked according to their positive distances (probabilities) to the trained SVM hyperplane. (2). To be more strictly, we also compared the *positive-samples-only-based manifold ranking *with one-class SVM algorithm presented by John B.S. et al. [22], with selected 10, 20 and 30 blood-secretory proteins as the positive samples. In such one-class SVM model, the whole test proteins were also ranked according to their positive distances (probabilities) to the trained SVM hyperplane.

(2). We then added the positive samples (253) and the negative samples (11,141) in the training set to the test set as queries to perform the *universal manifold ranking*. The idea is that we only kept the test data as an independent evaluation set. The samples in the training dataset were either used as labelled data to train the SVM for prediction on the test set, or used as queries to rank the samples in the test set. This is designed for a fair comparison between the two methods.

The performance by the *positive-samples-only-based manifold ranking *and SVM-based ranking on the independent test data is shown in Tables 1. It should be noted that in the first comparison strategy of SVM-based ranking, 250,130 and 110 times negative samples of the positive ones were heuristically selected for 10, 20 and 30 positive samples, to train an optimized SVM models, respectively. That is, 2,500, 2,600 and 3,300 negative samples were selected for 10, 20 and 30 positive samples incorporated with an exhaustive search of aforementioned in a range of [10:300] and the evaluations on the test dataset achieved the best accuracies at 98%, 99% and 99%, respectively. From Tables 1, we can see that the manifold ranking achieved the average best ranking result for all 3 groups of queries compared with both two kinds of SVM-based ranking.

**Table 1:** Performance comparisons on independent test dataset for manifold ranking and SVM-based ranking

*AUC*							
***No. of queries***	***Methods***	**1**	**2**	**3**	**4**	**5**	**Ave.**

**10**	**MR**	0.7412	0.6565	0.6342	0.6355	0.6576	0.6650
	
	**SVM-1**	0.6342	0.6224	0.6571	0.6317	0.5964	0.6284
	
	**SVM-2**	0.6425	0.6342	0.6521	0.6218	0.6091	0.6319

**20**	**MR**	0.7920	0.7629	0.7657	0.7574	0.8046	0.7765
	
	**SVM-1**	0.6768	0.6535	0.6373	0.6371	0.6895	0.6589
	
	**SVM-2**	0.6928	0.6634	0.6823	0.6576	0.6797	0.6752

**30**	**MR**	0.8245	0.8283	0.8170	0.8072	0.8655	0.8285
	
	**SVM-1**	0.7388	0.7800	0.8014	0.7864	0.7759	0.7765
	
	**SVM-2**	0.7818	0.8167	0.8023	0.7689	0.7909	0.7921

(Both rankings were performed with 10, 20 and 30 queries, evaluated by *AUC *of recall-precision curve. (Parameter setting: *σ *= 2.7003 and *α *= 0.5. Each kind of query is performed 5 times. MR: Manifold ranking; SVM-1: first strategy of SVM-based ranking; SVM-2: one-class SVM-based ranking)

**Figure 2:**
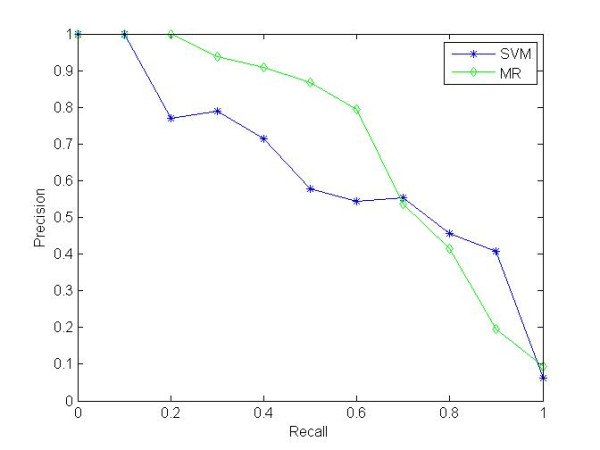
**Recall-precision curve of the ranking results on the whole test dataset given the whole training dataset as queries**.

Table 2 presents the performance on the test dataset based on the queries from training datasets, i.e., the SVM model is trained on the training datasets with 253 positive samples and 11,141 negative samples and the *universal manifold ranking *is performed with the same training samples as queries. A 10-fold cross-validation test has been performed and the SVM classifier can achieve ~98% accuracy on the test set. Here, we presented the general recall-precision curve on the whole test set with the whole training set as queries, which shows the corresponding prediction precision at each sensitivity (recall) level (Figure 2). It can be seen that, in this case, the universal manifold ranking method is superior to SVM-based ranking.

**Table 2:** Performance comparisons on independent test dataset for the universal manifold ranking and SVM-based ranking

	MR	SVM
***AUC***	0.6663	0.6592

(Both rankings were performed with a different dataset as training set, evaluated by *AUC *of recall-precision curve. Parameter setting: *σ *= 2.7003 and *α *= 0.5. MR: Manifold ranking; SVM: SVM-based ranking)

### Manifold ranking on all known human proteins

We have used the 305 known blood-secretory proteins as queries to perform our *positive-samples-only-based manifold ranking *of the whole set of 20,309 known human proteins collected from *Swissprot *[16]. The top-1,000 ranked proteins are given in additional file 2.

This ranking list can be viewed as a set of blood-secretory protein candidates for further experimental validations. Among these proteins, the top 305 ones are exactly the known blood-secreted proteins ordered by our ranking algorithm, while the other 695 proteins are originally considered as unknown but our prediction suggests that they are highly probable to be blood-secreted. Before the experimental validation on these predicted proteins, we indeed have collected some evidences to support some of our predictions. For example, protein *Cadherin-12 *(P55289), ranked as the 1st among the 695 proteins, has been recently identified to be a serum biomarker by multidimensional chromatography [2], while being currently annotated as a cell adhesion protein specifically expressed in brain tissue in *Swissprot *[16].

We have carried out a functional enrichment analysis on the top 1,000 proteins against the whole background human protein set, according to the GO ontology. The *GOEAST *(Gene Ontology Enrichment Analysis Software Toolkit) was used [23] to carry out this analysis. The comparison results in terms of three GO categories, *i.e*., biological process, cellular component and molecular function are listed in additional files 3, 4, 5. Since the GO ontology is hierarchical, we picked the top three most significantly enriched GO functional terms from the leaf nodes of each GO hierarchy, respectively, as listed in Table 3. We can see that these proteins are functionally enriched with serine endopeptidases, which are reported to serve functions in blood clotting, the immune system, and inflammation [24]. As expected, most of these proteins are membrane-associated proteins and related to platelet granule secretion [25]. There are literature evidences indicating that several serum biomarkers, such as the serum prostate specific antigens (PSA) for prostate cancer diagnosis, are shown to have higher levels of internal peptide bond cleavages and are more enzymatically inactive [26,27], thus it is particular interesting that we found the biological process of regulation of protein maturation by peptide bond cleavage is highly enriched in our dataset.

**Table 3:** Top 3 functional enrichment GO terms for the top 1,000 proteins provided by manifold ranking, annotated with 3 GO categories.

	molecular_function	cellular_component	biological_process
***Top 1***	GO:0004867: serine-type endopeptidase inhibitor activity (3.56e-27)	GO:0031093: platelet alpha granule lumen (1.64e-21)	GO:0010953: regulation of protein maturation by peptide bond cleavage (0)

***Top 2***	GO:0004252: serine-type endopeptidase activity (6.64e-14)	GO:0005606: laminin-1 complex (3.74e-8)	GO:0006958: complement activation, classical pathway (4.2e-21)

***Top 3***	GO:0008201: heparin binding (8.14e-8)	GO:0005579: membrane attack complex (3.74e-8)	GO:0006957: complement activation, alternative pathway (2.05e-16)

## Conclusion

In this study, we modelled the problem of blood-secretion prediction as a ranking instead of a classification problem, where novel blood-secretory proteins were ranked based on their relevance of a group of experimentally validated blood-secretory proteins. Our evaluation results have shown that the ranking algorithm is robust, efficient and achieved a superior prediction result than an SVM-based prediction method. We have presented the first time a complete blood-secretory protein ranking list on all human proteins, which is expected to well facilitate the experimental approach for serum biomarker discovery.

## Availability

The human protein dataset and the related scripts can be freely accessed at http://csbl.bmb.uga.edu/publications/materials/qiliu/blood_secretory_protein.html.

## Authors' contributions

QL carried out the design and implementation of the computational pipeline and drafted the manuscript. CJ was responsible for the compilation and analysis of data and participated in the revision of the manuscript. QY and YX conceived the study and coordinated the data analyses as well as revising the manuscript. All authors read and approved the final manuscript.

## Supplementary Material

Additional file 1 **Supplementary 1**. protein feature list. Click here for file

Additional file 2 **Supplementary 2**. top 1000 blood-secretory proteins predicted by manifold ranking. Click here for file

Additional file 3 **Supplementary 3**. Enrichment of Biological Processing GO annotation for top 1000 blood- secretory proteins predicted by manifold ranking. Click here for file

Additional file 4 **Supplementary 4**. Enrichment of Cellular Component GO annotation for top 1000 blood- secretory proteins predicted by manifold ranking. Click here for file

Additional file 5 **Supplementary 5**. Enrichment of Molecular Function GO annotation for top 1000 blood- secretory proteins predicted by manifold ranking. Click here for file
