# Supplementary figures and images for: In-silico prediction of blood-secretory human proteins using a ranking algorithm
Source: BMC Bioinformatics. 2010 May 14;11:250. doi: 10.1186/1471-2105-11-250 (PMC2877692; doi:10.1186/1471-2105-11-250)

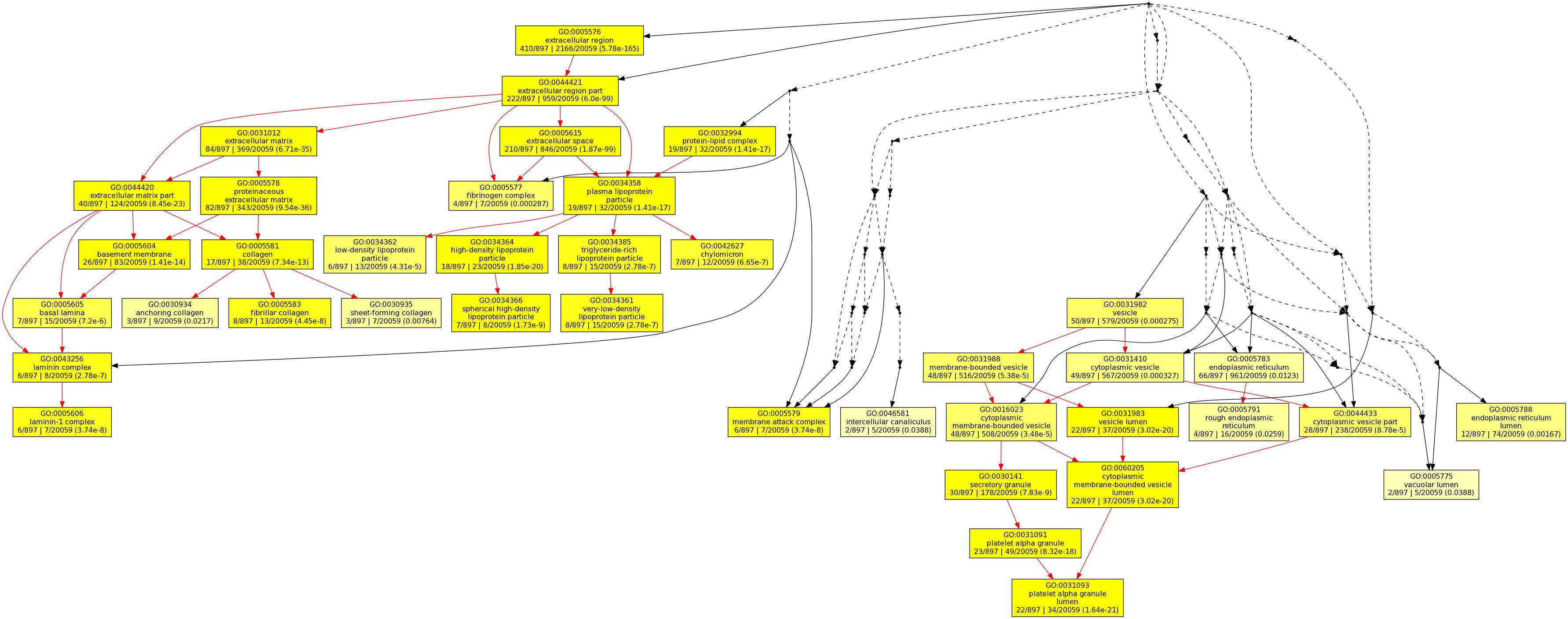

Supplement: Additional file 4 — Supplementary 4. Enrichment of Cellular Component GO annotation for top 1000 blood- secretory proteins predicted by manifold ranking. [file 1471-2105-11-250-S4.JPEG]

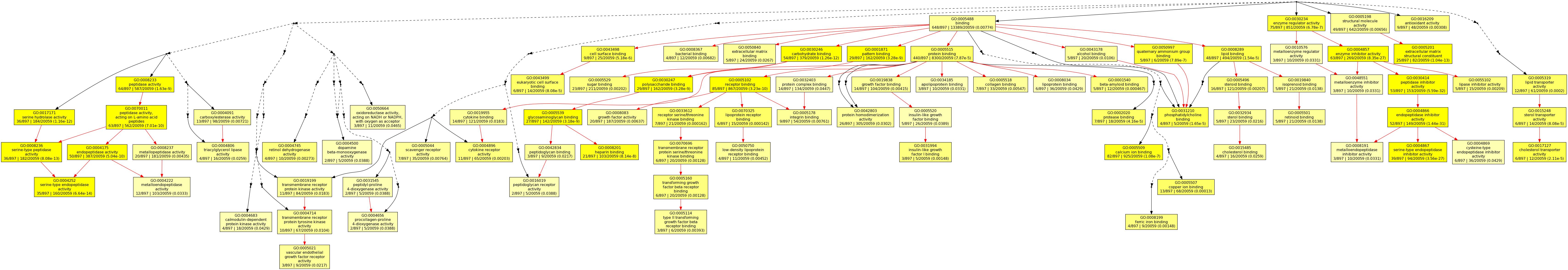

Supplement: Additional file 5 — Supplementary 5. Enrichment of Molecular Function GO annotation for top 1000 blood- secretory proteins predicted by manifold ranking. [file 1471-2105-11-250-S5.JPEG]
